# Dietitian-led individualized pancreatic enzyme replacement therapy is associated with favorable nutritional and immune-inflammatory profiles in pancreatic cancer: a retrospective cohort study

**DOI:** 10.3389/fnut.2026.1683101

**Published:** 2026-07-21

**Authors:** Xiaosu Chen, Junxuan Chen, Ting Zhu, Bing Xia, Guangjun Liu, Shu-an Wang, Junping Pan, Xiao-tian Chen

**Affiliations:** 1 Department of Clinical Nutrition, Nanjing Drum Tower Hospital, Affiliated Hospital of Medical School, Nanjing University, Nanjing, Jiangsu, China; 2 The Affiliated Drum Tower Hospital Clinical College of Xuzhou Medical University, Xuzhou, Jiangsu, China; 3 Department of Clinical Nutrition, Children’s Hospital of Nanjing Medical University, Nanjing, China; 4 Key Laboratory of Environmental Medicine and Engineering of Ministry of Education, Department of Nutrition and Food Hygiene, School of Public Health, Southeast University, Nanjing, Jiangsu, China; 5 The First Clinical Medical College, Xuzhou Medical University, Xuzhou, Jiangsu, China; 6 Division of Hepatobiliary and Liver Transplantation Surgery, Nanjing Drum Tower Hospital, Affiliated Hospital of Medical School, Nanjing University, Nanjing, Jiangsu, China

**Keywords:** body composition, immune-inflammatory biomarkers, nutritional counseling, pancreatic cancer, PERT

## Abstract

**Background:**

Pancreatic cancer is a highly malignant tumor with extremely poor prognosis. Pancreatic exocrine insufficiency (PEI), malnutrition, and immune-inflammatory dysfunction are highly prevalent and closely interact to impair postoperative recovery and clinical outcomes. Pancreatic enzyme replacement therapy (PERT) is the standard care for PEI; however, evidence regarding the association between dietitian-led individualized PERT and nutritional as well as immune-inflammatory profiles remains limited.

**Methods:**

This retrospective cohort study enrolled 123 patients with histologically confirmed pancreatic cancer who underwent surgical resection at Nanjing Drum Tower Hospital, affiliated to Nanjing University Medical School, from January 2018 to December 2023. Patients were retrospectively allocated to the Individualized PERT group (*n* = 58) and the Standard PERT group (*n* = 65) according to the type of PERT received in clinical practice. The Individualized PERT group received dietitian-led PERT combined with regular nutritional counseling, whereas the Standard PERT group received standard clinical PERT regimen combined with regular nutritional counseling. Baseline characteristics, nutritional parameters, body composition indices, hematological and immune-inflammatory biomarkers, as well as postoperative complications and 30-day readmission rates were collected and analyzed. Multivariable linear regression and Gamma generalized linear models (GLMs) were applied to adjust for confounding factors, including tumor stage, baseline hemoglobin and skeletal muscle index (SMI).

**Results:**

Dietitian-led individualized PERT was significantly associated with better preservation of body weight. The mean difference in weight change between the Individualized PERT and Standard PERT groups was 3.29 kg (95% CI: 1.32–5.27; *P* = 0.001). The corresponding mean percentage weight change was−1.53% ± 8.03% in the Individualized PERT group and−6.90% ± 9.07% in the Standard PERT group, with a mean difference of 5.37% (95% CI: 2.32%–8.43%; *P* < 0.001). No significant between-group difference was observed for serum albumin (*P* = 0.110), despite a positive trend. Body composition analysis confirmed that individualized PERT was associated with milder loss of skeletal muscle mass and adipose tissue. Furthermore, key immune-inflammatory biomarkers, including lymphocyte count and the lymphocyte-C-reactive protein ratio, showed more favorable levels in the Individualized PERT group. After multivariate adjustment, individualized PERT remained independently associated with improved body weight, body mass index (BMI), SMI, reduced C-reactive protein-to-albumin ratio (CAR) and higher LCR. There were no significant differences between the groups in the rates of postoperative complications (*P* = 0.588) or 30-day readmission (*P* = 0.326).

**Conclusion:**

In this retrospective cohort study, dietitian-led individualized PERT combined with nutritional counseling is independently associated with favorable nutritional and immune-inflammatory profiles among pancreatic cancer patients with PEI after adjusting for major confounders.

## 1 Introduction

### 1.1 Background

Pancreatic cancer is an exceptionally lethal and aggressive malignancy, with a 5-year survival rate of only 13% in the United States according to statistics from the American Cancer Society (1, 2). The incidence of pancreatic cancer is on the rise globally (3). Due to the unique anatomical location and biological characteristics of pancreatic cancer, most patients are diagnosed at an advanced stage, by which time the optimal opportunity for radical surgical intervention has often elapsed (1). Even when certain patients qualify for surgical intervention, they often face a variety of complex postoperative complications, notably including malnutrition and immune dysfunction (1, 4–7). Malnutrition is highly prevalent among patients with pancreatic cancer (8, 9). This condition arises not only due to the metabolic demands of the tumor but is also closely associated with the impaired digestive and absorptive functions that result from the disease (10, 11). The pancreas, a vital organ in the human digestive system, primarily secretes various digestive enzymes that facilitate nutrient absorption (12, 13). When pancreatic function is compromised, patients may present with loss of appetite, indigestion, and weight loss, which can lead to severe malnutrition (11, 14, 15). Pancreatic exocrine insufficiency (PEI) is a condition characterized by the pancreas’s inability to secrete sufficient amounts of specific digestive enzymes into the small intestine. This enzymatic deficiency impairs the absorption of nutrients, which may result in varying degrees of nutritional deficiencies (11). Moreover, immune dysfunction poses a substantial challenge for patients with pancreatic cancer (4). Malnutrition exacerbates immune impairment, heightens susceptibility to infections, and can result in various complications, all of which adversely affect treatment outcomes and diminish patients’ quality of life (16, 17).

### 1.2 Rationale and knowledge gap

Currently, the treatment options available for malnutrition and immune dysfunction in patients with pancreatic cancer are limited, often resulting in suboptimal clinical outcomes. Pancreatic enzyme replacement therapy (PERT) has received increasing attention in recent years for managing PEI and improving nutrient absorption in pancreatic cancer patients (11, 18). However, large-scale clinical studies investigating the association between PERT and nutritional as well as immune status among pancreatic cancer patients remain scarce.

### 1.3 Objective

Pancreatic enzyme replacement therapy has long been recognized as the standard treatment for PEI in clinical practice (11). However, the inappropriate application of pancreatic enzyme supplements remains a prevalent issue in clinical settings, adversely affecting treatment efficacy (19, 20). Therefore, this retrospective cohort study compared nutritional parameters, body composition indices, hematological and immune-inflammatory biomarkers, as well as postoperative complications and 30-day readmission rates between pancreatic cancer patients receiving dietitian-led individualized PERT and those receiving standard PERT, aiming to explore the association between dietitian-led individualized PERT and clinical indicators including nutrition, body composition and immune-inflammatory status.

## 2 Materials and methods

### 2.1 Research population and ethics

This retrospective cohort study analyzed clinical data retrieved from the electronic medical records of patients who received pancreatic surgery at Nanjing Drum Tower Hospital, Affiliated Hospital of Nanjing University Medical School, from January 2018 to December 2023. The study was conducted in accordance with the Declaration of Helsinki (2013 revision) and approved by the Ethics Committee of Nanjing Drum Tower Hospital (Approval No. 2024-558-02). Informed consent was waived due to the retrospective, de-identified design, in compliance with national ethical guidelines for biomedical research involving human beings.

Inclusion criteria:

(1) Histologically confirmed pancreatic cancer per NCCN guidelines.
(2) Underwent surgical resection at our institution.
(3) Age ≥ 18 years.
(4) A score of ≥3 on the Nutrition Risk Screening 2002 (NRS-2002) scale (21).
(5) PEI was diagnosed in accordance with the Chinese guidelines for the diagnosis and treatment of pancreatic exocrine insufficiency (2018 edition) (22). Diagnosis was established if any of the following criteria were met: ➀ Fecal elastase-1 (FE-1) test results indicate <200 μg/g of stool; ➁ A 72-hour quantitative fecal fat test reveals steatorrhea, defined as fecal fat content exceeding 7 g/24 h; ➂ Typical symptoms of steatorrhea and ≥5% unintentional weight loss within 3 months preoperatively were documented, with obvious favorable changes after PERT. Steatorrhea was defined as ≥2 of the following: loose, greasy, foamy, foul-smelling, or floating stools.

Exclusion criteria:

(1) Severe organ dysfunction.

(2) Communication disorders or a prior history of psychiatric illness.

(3) Vegetarian diet or selective eating behavior.

(4) Known hypersensitivity to enteric-coated pancreatic enzyme capsules (Creon^®^).

(5) Other conditions deemed unsuitable for study participation by investigators.

### 2.2 Research design and group allocation

**FIGURE 1:**
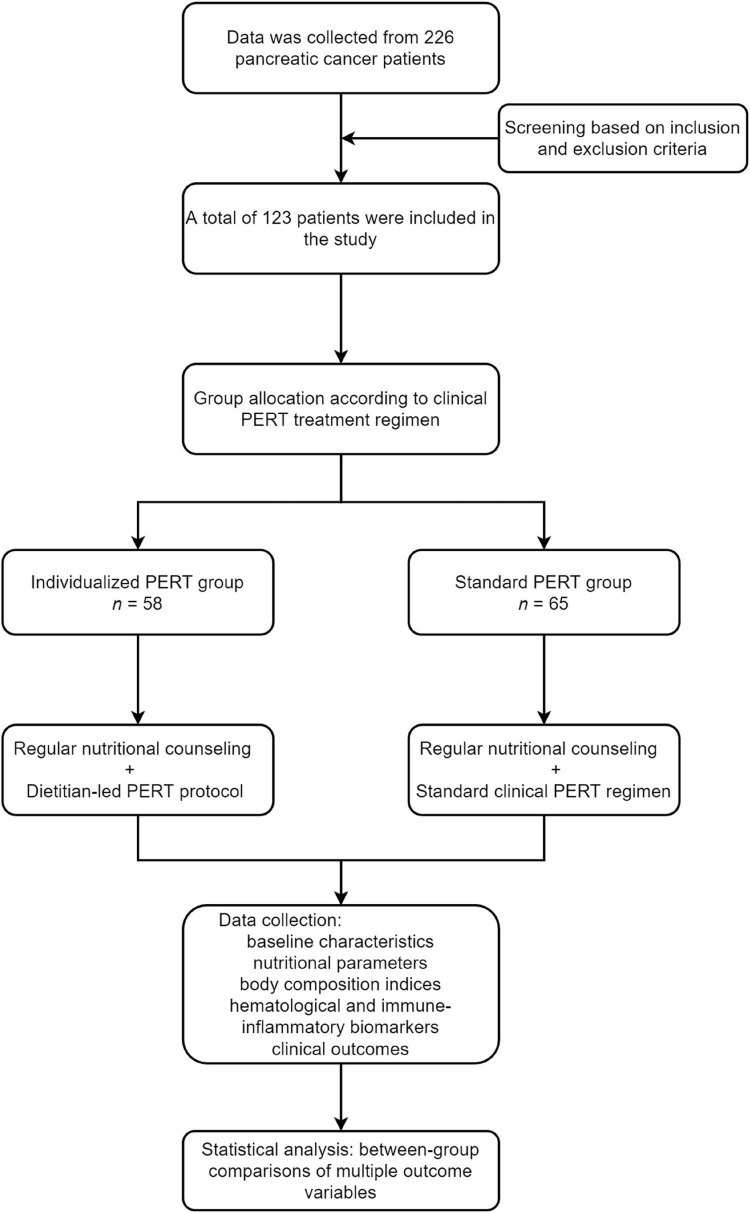
Flowchart of patient enrollment and study design.

Patients were divided into two groups based on the PERT regimen administered in clinical practice: (1) Individualized PERT group (*n* = 58): regular nutritional counseling + dietitian-designed, titrated, and supervised individualized PERT; (2) Standard PERT group (*n* = 65): regular nutritional counseling + standard clinical PERT per institutional guidelines. The patient enrollment flowchart is shown in Figure 1.

### 2.3 Surgical treatment

According to the Chinese Society of Clinical Oncology (CSCO): Clinical guidelines for the diagnosis and treatment of pancreatic cancer (2022 edition) (23), all surgical procedures for study participants were performed by experienced pancreatic surgeons in strict accordance with these guidelines. Standard radical procedures included pancreaticoduodenectomy, distal pancreatectomy, perineural resection, total splenectomy, and abdominal lymph node dissection. Before surgery, a comprehensive evaluation of each patient’s nutritional status, tumor stage, and the anatomical relationship between the lesion and surrounding tissues and organs was performed to formulate an appropriate surgical plan. During the operation, the resection criteria outlined in the guidelines were rigorously followed to ensure precise removal of relevant structures. Vascular management and critical anastomotic steps were implemented in compliance with the guidelines to minimize the risk of postoperative complications.

### 2.4 Regular nutritional counseling

Regular nutritional counseling was provided by a qualified dietitian to all patients in both groups. During hospitalization, the dietitian delivered the counseling via face-to-face instruction, supplemented by written dietary guidance before discharge. The plan was developed based on the Expert Consensus on Home Medical Care and Home Nursing Management for Pancreatic Cancer Patients in China (6). The primary objective was to maintain daily energy intake at 25–30 kcal/kg, with a protein intake of 1.2–2.0 g/kg. For patients with complications such as infection or intestinal fistula, the recommended energy intake was adjusted to 30–35 kcal/kg. The prescribed dietary types encompassed soft, semi-liquid, and liquid diets, with specific restrictions on alcohol consumption, fatty meats, and processed meat products. Preferred cooking methods included steaming, boiling, stewing, poaching, and simmering, while frying, deep-frying, roasting, smoking, and pickling were discouraged. During the postoperative fasting period, total parenteral nutrition support was provided, followed by a gradual transition to a regular diet prior to discharge.

### 2.5 PERT treatment

Exogenous digestive enzymes (commercial name: Creon ^®^; specification: each capsule containing 10,000 units of lipase, 8,000 units of pancreatic amylase, and 600 units of trypsin) were administered as part of the treatment regimen.

#### 2.5.1 Standard clinical PERT regimen (standard PERT group)

Based on regular nutritional counseling, patients with PEI received routine PERT: 2 capsules each time, 30 min before meals, three times per day. The dosage was adjusted to 3 capsules per time only when obvious steatorrhea (fecal fat > 15 g/d) was present. No fixed monitoring frequency or quantitative adjustment criteria were applied.

#### 2.5.2 Dietitian-led individualized PERT protocol (individualized PERT group)

On the basis of regular nutritional counseling, patients received an individualized PERT protocol designed, titrated, and supervised by a dietitian. The initial dose range was set at 2–4 capsules with main meals and 1–2 capsules with snacks or oral nutritional supplements. The exact number of capsules within each range was determined individually by the dietitian according to the patient’s meal-specific fat intake, body weight, and severity of steatorrhea, and was clearly documented in the patient’s dietary guidance sheet. All patients received standardized dietary education and detailed guidance from the dietitian regarding enzyme administration with meals, dose titration within the recommended range, and recognition of symptoms necessitating further dose adjustment. Patients were instructed to take capsules with meals to minimize the interval between enzyme administration and food intake. If symptoms showed no favorable changes after the dose reached 4 capsules per main meal (particularly steatorrhea ≥2 times per week), a proton pump inhibitor, such as omeprazole at 40 mg per day, was added.

### 2.6 Observation indicators

#### 2.6.1 Baseline demographic and clinical characteristics

Baseline characteristics were collected from electronic medical records of patients, including age, sex, height, weight, BMI, family history, surgical procedure, tumor stage [classified according to the 8th edition of the American Joint Committee on Cancer (AJCC) TNM staging system for pancreatic cancer], and pathological classification.

#### 2.6.2 Hematological and immune-inflammatory biomarkers

Hematological and immune-inflammatory biomarkers were collected from electronic medical records, including serum albumin (ALB), hemoglobin (HB), white blood cell count (WBC), lymphocyte count (LBC), neutrophil count (NC), platelet count (PC), monocyte count (MC), C-reactive protein (CRP), procalcitonin (PCT), and interleukin-6 (IL-6).

Based on these biomarkers, a panel of composite immune-inflammatory biomarkers was calculated. These combined biomarkers are widely validated in pancreatic cancer and surgical nutrition research to reflect systemic immune function, inflammatory burden, and immunonutritional status (24–27).

The composite immune-inflammatory biomarkers included: C-reactive protein-to-albumin ratio (CAR), lymphocyte CRP score (LCS), lymphocyte-to-monocyte ratio (LMR), cachexia index (CXI), neutrophil-lymphocyte ratio (NLR), platelet-lymphocyte ratio (PLR), and lymphocyte-C-reactive protein ratio (LCR). The specific calculation methods are as follows (26, 28–30):

LCS: A score of 0 was assigned when LBC ≥ 1 × 10^9^/L and CRP ≤ 3.0 mg/L; a score of 1 was assigned when LBC < 1 × 10^9^/L or CRP > 3.0 mg/L; a score of 2 was assigned when LBC < 1 × 10^9^/L and CRP > 3.0 mg/L.

CAR = CRP (mg/L) ÷ ALB (g/L)

LMR = LBC (×10^9^/L)÷MC (×10^9^/L)

NLR = NC (×10^9^/L) ÷ LBC (×10^9^/L)

PLR = PC (×10^9^/L) ÷ LBC (×10^9^/L)

LCR = LBC (×10^9^/L) ÷ CRP (mg/L)

CXI = (SMI × ALB)/NLR

#### 2.6.3 Body composition indices

**FIGURE 2:**
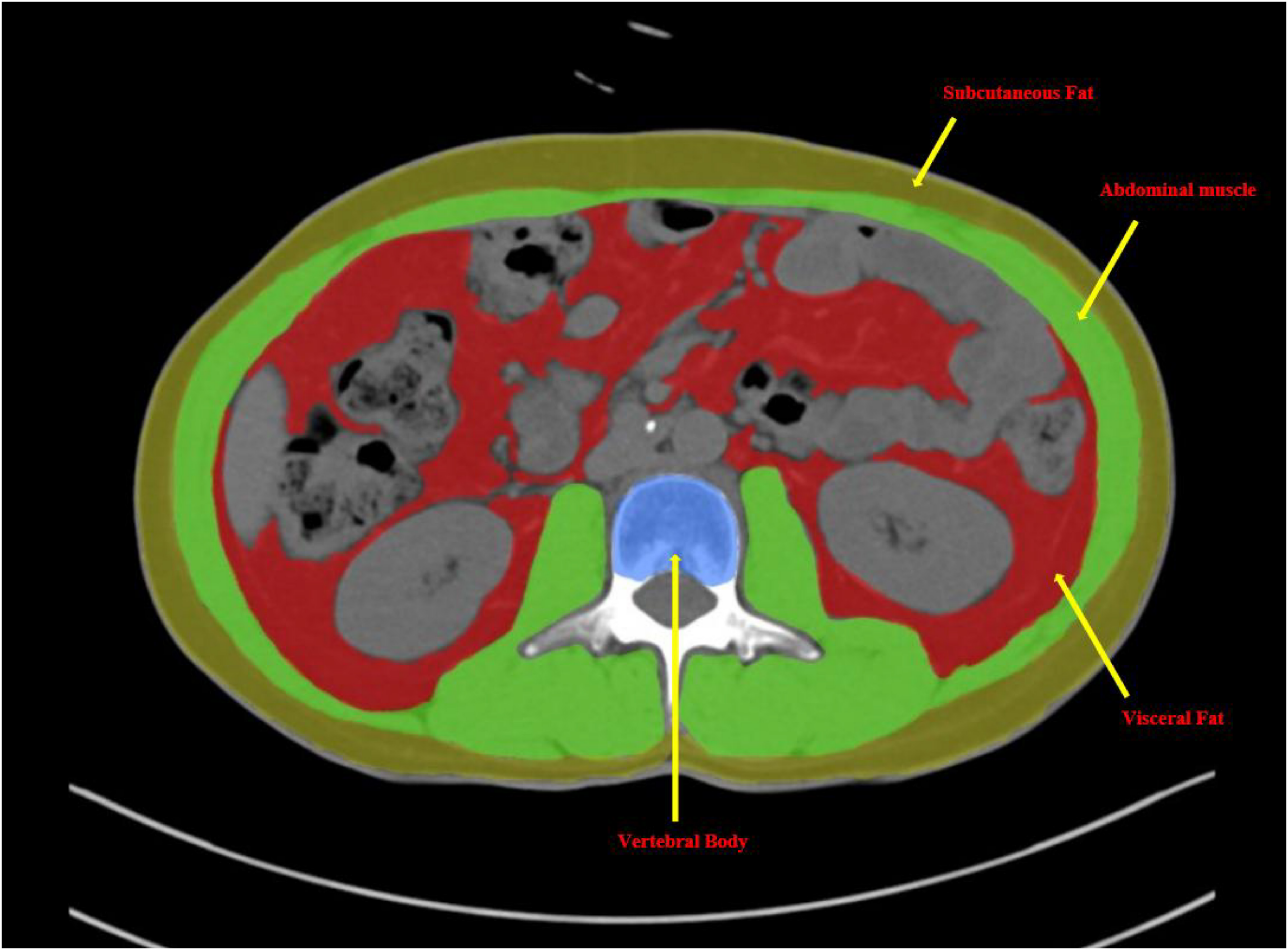
Representative axial abdominal computed tomography (CT) image at the L3 level with segmentation of body composition components. The yellow area indicates subcutaneous adipose tissue; the green area indicates abdominal skeletal muscle (including psoas major, erector spinae, quadratus lumborum, transversus abdominis, internal oblique, external oblique, and rectus abdominis); the red area indicates visceral adipose tissue; the blue area indicates the vertebral body. Yellow arrows highlight each color-coded component.

Imaging data were collected from patients. Axial computed tomography (CT) images were obtained from the Picture Archiving and Communication System (PACS) system of the Department of Medical Imaging, Nanjing Drum Tower Hospital, Affiliated Hospital of Nanjing University Medical School. Using AccuContour software, manual anatomical tracing (segmentation) was performed on axial CT images at the L3 level to identify and quantify skeletal muscle, visceral adipose tissue, subcutaneous adipose tissue, and vertebral bone density (Figure 2).

The skeletal muscle at the L3 level included the psoas major, erector spinae, quadratus lumborum, transversus abdominis, internal and external oblique muscles, and rectus abdominis. Visceral adipose tissue area (VAT), subcutaneous adipose tissue area (SAT), skeletal muscle area (SMA), and skeletal muscle density (SMD) were measured, and skeletal muscle index (SMI, cm^2^/m^2^) was calculated by dividing the skeletal muscle area (SMA, cm^2^) measured at the L3 level by the square of body height (m^2^): SMI = SMA/height^2^. All CT body composition analyses were performed independently by a single experienced radiologist who was blinded to group assignment to avoid measurement bias.

#### 2.6.4 Clinical outcomes

Postoperative complications and 30-day readmission data were obtained from electronic medical records. Readmission was defined as unplanned rehospitalization for treatment or observation within 30 days after discharge due to operation-related causes.

#### 2.6.5 Timing of data collection

All data were collected at two time points: at surgical admission before operation (baseline) and prior to the first cycle of chemotherapy (endpoint), with a median interval of 30 days (range 21–45 days) between the two time points.

### 2.7 Primary and secondary outcomes

The primary outcome was the between-group difference in body weight change from baseline to endpoint. Secondary outcomes included serum ALB level, body composition indices (SMI, SMA, SAT, VAT, etc.), immune-inflammatory biomarkers (CRP, CAR, LCR, LCS, NLR, PLR, LMR, CXI, etc.), postoperative complications and 30-day readmission rates.

### 2.8 Statistical analysis

Baseline characteristics, body composition indices, hematological and immune-inflammatory biomarkers, and clinical outcomes were compared between the Individualized PERT group and Standard PERT group. Additionally, postoperative complications and 30-day readmission rates were compared between the two groups. SPSS version 27.0 software was used for statistical analysis. Shapiro-Wilk test was used to check for normality. For normally distributed data, mean ± standard deviation was used, and *t*-tests were employed for inter-group comparisons. For non-normally distributed data, median (interquartile range, IQR, P25, P75) was used, and Mann-Whitney U tests were used for inter-group comparisons. Categorical data were presented as frequencies, rates, or percentages (%), and χ^2^ tests were used for inter-group comparisons. A *P*-value < 0.05 indicated statistically significant differences.

To adjust for confounders, multivariable linear regression was applied for normally distributed continuous outcomes, and Gamma generalized linear models (GLMs) with a log link for skewed variables (Supplementary Table 1). Covariates were selected based on baseline imbalance, clinical plausibility, and avoidance of overfitting. Tumor stage (dichotomized as ≤ 1 vs. >1), baseline HB and SMI were included in models, as all are established determinants of nutritional and inflammatory status in pancreatic cancer and showed baseline between-group differences (31, 32).

## 3 Results

### 3.1 Baseline demographic and clinical characteristics of the study subjects

A total of 226 patients were initially screened for eligibility. After applying inclusion and exclusion criteria, 123 patients were finally included in the analysis, including 78 males and 45 females. The Individualized PERT group comprised 58 patients, and the Standard PERT group comprised 65 patients (Table 1). There were no significant between-group differences in age, sex, height, body weight, BMI, family history of cancer, surgical procedure type, or pathological classification (all *P* > 0.05) (Table 1). However, tumor stage differed significantly between the two groups at baseline (*P* = 0.025) (Table 1). This imbalance was adjusted for in subsequent multivariable analyses to reduce potential confounding (Supplementary Table 1). Overall, the two groups were generally comparable at baseline, supporting the validity of further between-group comparisons.

**TABLE 1:** Baseline demographic and clinical characteristics of study participants.

Characteristics	Standard PERT group (*n* = 65)	Individualized PERT group (*n* = 58)	*P*-value
Age (years)	66.53 ± 9.06	64.05 ± 8.06	0.111
Gender			0.405
Male (%)	39 (60.00)	39 (67.24)	
Female (%)	26 (40.00)	19 (32.76)	
Height (m)	1.66 ± 0.07	1.66 ± 0.09	0.788
Weight (kg)	61.11 ± 10.10	62.75 ± 9.93	0.364
BMI (kg/m^2^)	22.13 ± 3.12	22.87 ± 3.19	0.196
Family history			0.131
Yes (%)	0 (0.00)	2 (3.45)	
No (%)	65 (100.00)	56 (96.55)	
Surgical method			0.056
Pancreaticoduodenectomy (%)	49 (75.38)	34 (58.62)	
Others (%)	16 (24.62)	24 (41.38)	
Tumor stage			0.025
Stage I	25 (38.46)	34 (58.62)	
Stage II–IV	40 (61.54)	24 (41.38)	
Classification			0.414
Adenocarcinoma (%)	60 (92.31)	51 (87.93)	
Others (%)	5 (7.69)	7 (12.07)	

BMI, body mass index. Other surgical methods: Distal pancreatectomy, total splenectomy, and regional lymph node dissection. Other pathological classifications: Non-adenocarcinoma histologic types, including neuroendocrine neoplasms and other rare pancreatic tumor subtypes.

### 3.2 Primary outcome

Analysis of primary endpoint showed that the Individualized PERT group had significantly smaller body weight loss than the Standard PERT group (mean difference: 3.29 kg; 95% CI: 1.32–5.27; *P* = 0.001) (Table 2). The mean percentage weight change from baseline to endpoint was −1.53% ± 8.03% in the Individualized PERT group and −6.90% ± 9.07% in the Standard PERT group, with a mean difference of 5.37% (95% CI: 2.32%–8.43%; *P* < 0.001).

**TABLE 2:** Changes in nutritional parameters from baseline to endpoint (pre-first chemotherapy) in the two groups.

Variables	Time point	Standard PERT group (*n* = 65)	Individualized PERT group (*n* = 58)	Mean / median difference	Difference (95% CI)	t / *Z*-value	*P*-value
Weight (kg)							
	Base line	61.11 ± 10.10	62.75 ± 9.93	1.64	(−1.92∼ 5.20)	0.91	0.364
	Endpoint	56.53 ± 8.85	61.46 ± 8.73	4.93	(1.80∼ 8.06)	3.12	0.002
	Δ value	−4.58 ± 6.17	−1.29 ± 4.81	3.29	(1.32∼ 5.27)	3.30	0.001
Weight change percentage (%)		−6.90 ± 9.07	−1.53 ± 8.03	5.37	(2.32∼ 8.43)	3.48	<0.001
BMI (kg/m2)							
	Base line	22.13 ± 3.12	22.87 ± 3.19	0.74	(−0.39∼ 1.86)	1.30	0.196
	Endpoint	20.43 ± 2.31	22.41 ± 2.86	1.98	(1.06∼−2.90)	4.26	<0.001
	Δ value	−1.70 ± 2.36	−0.46 ± 1.82	1.24	(0.49∼ 1.99)	3.27	0.001
ALB (g/L)							
	Base line	39.06 ± 3.16	39.78 ± 2.40	0.72	(−0.29∼ 1.72)	1.41	0.160
	Endpoint	34.80 ± 3.93	36.87 ± 3.93	2.06	(0.67∼ 3.46)	2.92	0.004
	Δ value	−4.26 ± 4.33	−2.91 ± 4.99	1.35	(0.31 ∼ 3.01)	1.61	0.110
HB (g/L)							
	Base line	125.20 ± 14.86	131.00 ± 13.17	5.80	(0.79 ∼ 10.80)	2.29	0.024
	Endpoint	104.39 ± 19.80	110.03 ± 17.70	5.64	(−1.05 ∼ 12.33)	1.67	0.098
	Δ value	−20.81 ± 19.78	−20.97 ± 17.88	−0.16	(−6.88 ∼ 6.56)	−0.05	0.963
CXI							
	Base line	619.00 (459.20, 876.54)	666.63 (416.07, 983.74)	29.95	(−102.54 ∼ 156.73)	−0.41	0.682
	Endpoint	162.28 (91.75, 462.12)	257.85 (156.08, 574.99)	74.79	(14.39 ∼ 136.01)	−2.36	0.019
	Δ value	−400.59 (−594.12, −181.85)	−292.40 (−603.21, −88.95)	67.90	(−75.10 ∼ 207.18)	−1.02	0.310

Baseline was defined as preoperative assessment at surgical admission; endpoint was defined as prior to the first cycle of chemotherapy, with a median interval of 30 days (range 21–45 days) between the two time points. Normal distribution data presented as mean ± standard deviation (SD); between-group difference reported as mean difference with 95% CI of mean difference; non-normal distribution data presented as median (interquartile range, IQR); between-group difference reported as median difference with 95% CI for median; *P*-value for between-group comparison of changes. Multivariable adjusted results are presented in Supplementary Table 1. CI, confidence interval; BMI, body mass index; ALB, albumin; HB, hemoglobin; CXI, cachexia index; Δ value, the difference between the endpoint value and baseline value for each variable.

### 3.3 Secondary outcomes

#### 3.3.1 Changes in nutritional parameters

At baseline, there were no significant between-group differences in BMI or CXI. HB levels were significantly higher in the intervention group at baseline (Table 2). At the endpoint, BMI and CXI were significantly higher in the Individualized PERT group, and the reduction in BMI was significantly smaller in this group. Although serum ALB did not show a significant between-group difference in its change over time, the Individualized PERT group exhibited a smaller reduction. The change in HB did not differ significantly between groups (Table 2). These findings suggest that the individualized PERT was correlated with slower deterioration of nutritional status.

#### 3.3.2 Changes in body composition indices

At baseline, SMD, SMA, SAT, and VAT were comparable between groups, whereas SMI was significantly higher in the Individualized PERT group (Supplementary Table 2). At the endpoint, SMD did not differ significantly between groups, but its reduction was greater in the Individualized PERT group. In contrast, SMA, SMI, SAT, and VAT were all significantly higher in the Individualized PERT group. Changes from baseline to endpoint confirmed that individualized PERT was associated with milder losses of skeletal muscle mass and adipose tissue (Supplementary Table 2). L3 vertebral bone density did not differ significantly between groups at either time point.

#### 3.3.3 Changes in immune biomarkers

White blood cell count, NC, NLR, and PLR were comparable between groups at both time points, and their changes were not significantly different (Table 3). LBC was similar at baseline and endpoint, but the Individualized PERT group showed a significantly smaller reduction in LBC over time. LCR was comparable at baseline and significantly higher in the Individualized PERT group at the endpoint (Table 3). These findings indicate that the two groups differed in lymphocyte-mediated immune function, with more favorable results in the individualized PERT group.

**TABLE 3:** Changes in immune biomarkers from baseline to endpoint.

Variables	Time point	Standard PERT group (*n* = 65)	Individualized PERT group (*n* = 58)	Mean / median difference	Difference (95% CI)	*Z*-value	*P*-value
WBC (×109/L)							
	Baseline	5.50 (4.50, 6.70)	5.40 (4.70, 6.18)	−0.10	(−0.70 ∼ 0.40)	−0.47	0.640
	Endpoint	8.60 (5.70, 13.40)	8.85 (6.63, 12.55)	0.70	(−0.90 ∼ 2.10)	−0.81	0.416
	Δ value	2.40 (0.30, 5.40)	4.20 (0.55, 7.40)	0.90	(−0.60 ∼ 2.50)	−1.15	0.252
LBC (×109/L)							
	Baseline	1.50 (1.10, 1.90)	1.40 (1.00, 1.98)	−0.10	(−0.30 ∼ 0.20)	−0.56	0.573
	Endpoint	1.10 (0.80, 1.40)	1.20 (0.93, 1.70)	0.20	(0.00 ∼ 0.30)	−1.78	0.075
	Δ value	−0.40 (−0.80, 0.00)	−0.10 (−0.50, 0.30)	0.30	(−0.10 ∼ 0.50)	−2.62	0.009
NC (×109/L)							
	Baseline	3.40 (2.70, 4.40)	3.40 (2.70, 4.48)	0.00	(−0.40 ∼ 0.40)	−0.10	0.922
	Endpoint	6.50 (3.60, 10.70)	7.30 (4.38, 10.03)	0.40	(−1.00 ∼ 1.80)	−0.64	0.522
	Δ value	2.50 (0.10, 5.40)	3.55 (0.60, 6.95)	0.60	(−0.90 ∼ 2.10)	−0.91	0.361
NLR							
	Baseline	2.29 (1.59, 3.22)	2.37 (1.68, 3.43)	0.10	(−0.28 ∼ 0.47)	−0.55	0.586
	Endpoint	5.93 (2.60, 11.00)	6.05 (2.86, 9.05)	−0.29	(−1.97 ∼ 1.15)	−0.47	0.640
	Δ value	3.21 (0.18, 8.26)	2.78 (0.36, 5.88)	−0.48	(−2.24 ∼ 1.07)	−0.67	0.503
PLR							
	Baseline	128.00 (93.33, 180.63)	119.17 (97.00, 192.13)	0.40	(−21.05 ∼ 19.23)	−0.03	0.980
	Endpoint	200.00 (146.67, 253.33)	171.61 (113.10, 291.46)	−20.89	(−56.36 ∼ 20.56)	−1.00	0.316
	Δ value	59.90 (−10.00, 123.08)	49.32 (−24.36, 151.05)	−12.51	(−50.65 ∼ 25.11)	−0.80	0.424
LCR							
	Baseline	0.38 (0.16, 0.67)	0.44 (0.22, 0.61)	0.01	(−0.10 ∼ 0.13)	−0.26	0.793
	Endpoint	0.04 (0.01, 0.21)	0.07 (0.03, 0.48)	0.02	(0.01 ∼ 0.06)	−2.60	0.009
	Δ value	−0.28 (−0.58, −0.05)	−0.24 (−0.53, 0.14)	0.10	(−0.08 ∼ 0.32)	−1.09	0.275

Normal distribution data presented as mean ± standard deviation (SD); between-group difference reported as mean difference with 95% CI of mean difference; non-normal distribution data presented as median (interquartile range, IQR); between-group difference reported as median difference with 95% CI for median; *P*-value for between-group comparison of changes. CI, confidence interval; WBC, white blood cell count; LBC, lymphocyte count; NC, neutrophil count; NLR, neutrophil - lymphocyte ratio; PLR, platelet - lymphocyte ratio; LCR, lymphocyte - C - reactive protein ratio; Δ value, the difference between the endpoint value and baseline value for each variable. All immune-inflammatory indices were calculated as described in section “2.6.2 Hematological and immune-inflammatory biomarkers.”

#### 3.3.4 Changes in inflammatory biomarkers

At baseline, all inflammatory markers were comparable between groups (Supplementary Table 3). At the endpoint, the Individualized PERT group exhibited significantly lower levels of CRP, CAR, and LCS. Changes from baseline further confirmed that the Individualized PERT group had significantly smaller increases in CAR and LCS (Supplementary Table 3). No significant between-group differences were observed for PCT, IL-6, or LMR.

#### 3.3.5 Comparison of postoperative complications and 30-day readmission

**FIGURE 3:**
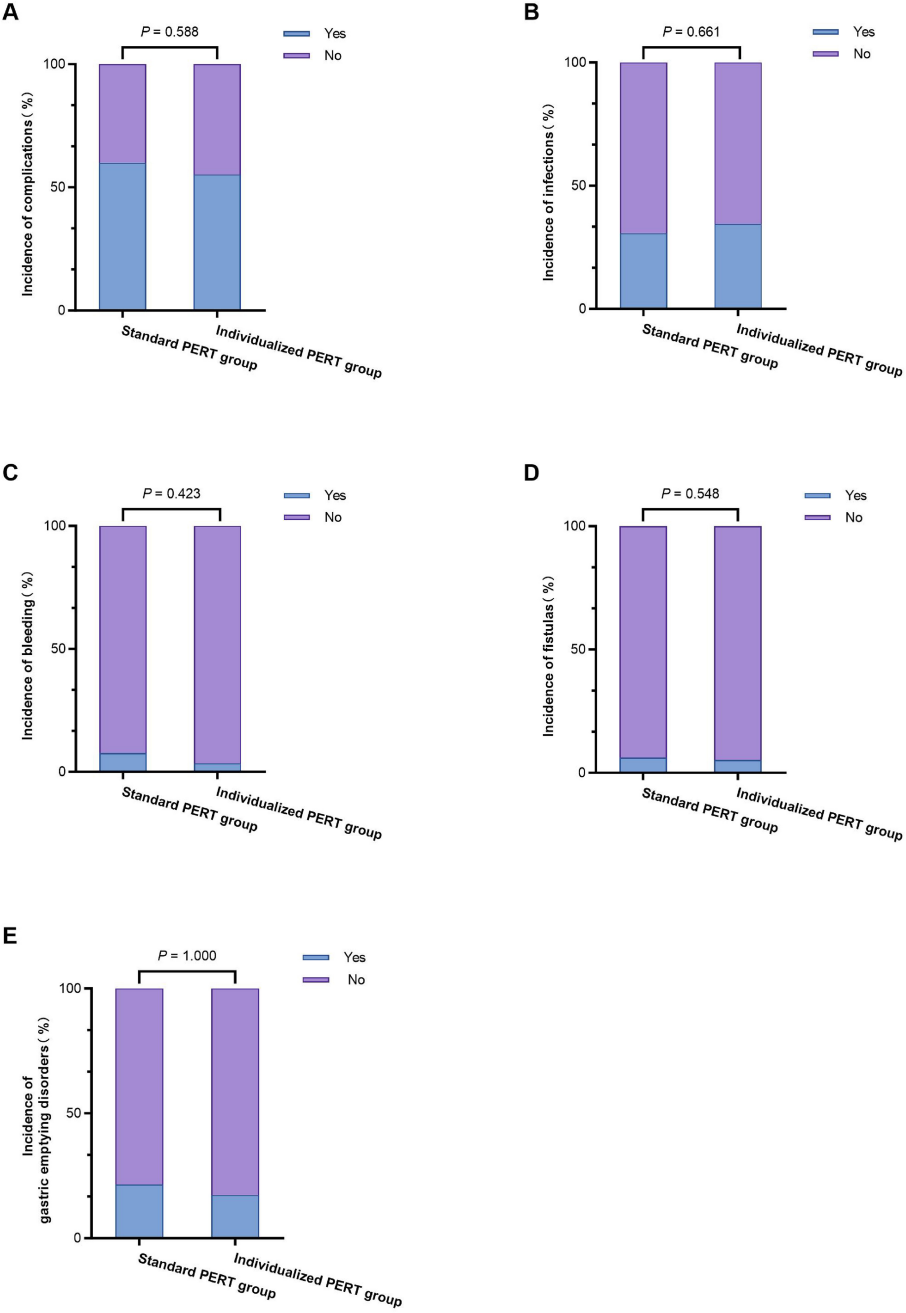
Postoperative complications in the two groups. **(A)** Overall incidence of postoperative complications; **(B)** incidence of infectious complications; **(C)** incidence of bleeding; **(D)** incidence of fistulas; **(E)** incidence of gastric emptying disorders.

The overall incidence of postoperative complications was comparable between the Individualized PERT and Standard PERT groups (*P* = 0.588) (Figure 3 and Table 4). No significant between-group differences were observed for individual complication types, including infectious complications, bleeding, fistula formation, or delayed gastric emptying (all *P* > 0.05) (Figure 3). The 30-day readmission rate was 4/58 (6.9%) in the Individualized PERT group and 2/65 (3.1%) in the Standard PERT group, with no statistically significant difference between groups (*P* = 0.326) (Table 4). These results indicate that dietitian-led individualized PERT was not associated with an elevated risk of postoperative complications or 30 - day readmission in patients undergoing pancreatic cancer surgery, supporting its short-term safety and clinical feasibility.

**TABLE 4:** Comparison of 30-day readmission rates between groups.

Variables	Standard PERT group (*n* = 65)	Individualized PERT group (*n* = 58)	χ^2^ value	*P*-value
Readmission after 30 days			0.96	0.326
No	63	54		
Yes	2	4		

#### 3.3.6 Adjusted associations between the individualized PERT and outcomes

To account for potential confounding factors, multivariable linear regression models were fitted for continuous outcomes with normal distribution, and Gamma generalized linear models (GLMs) with log link were applied for skewed continuous outcomes. All models were adjusted for tumor stage (dichotomized as ≤ 1 vs. >1), baseline HB, and baseline SMI, with the exception of the SMI change model, which was adjusted for tumor stage and baseline HB only (Supplementary Table 1).

After multivariable adjustment, individualized PERT was independently associated with significantly better weight preservation (β = 3.65, 95% CI 1.57–5.73, *P* < 0.001), reduced BMI loss (β = 1.36, 95% CI 0.56–2.15, *P* < 0.001), and attenuated SMI decline (β = 6.85, 95% CI 5.24–8.45, *P* < 0.001). For inflammatory biomarkers, individualized PERT was independently associated with a lower CAR (Exp(β) = 0.50, 95% CI 0.34–0.74, *P* < 0.001) and higher LCR (Exp(β) = 2.52, 95% CI 1.10–5.75, *P* = 0.028).

## 4 Discussion

In this retrospective cohort study, dietitian-led individualized PERT combined with nutritional counseling was observed to be associated with preserved body weight, skeletal muscle mass, and adipose tissue, and more favorable immune-inflammatory status, and these beneficial associations remained significant after multivariate adjustment for confounders. Meanwhile, individualized PERT showed acceptable short-term safety in postoperative pancreatic cancer patients with PEI.

The primary endpoint of this study was the between-group difference in body weight change from baseline to the endpoint. The Individualized PERT group exhibited significantly less weight loss than the Standard PERT group, with a mean difference of 3.29 kg (95% CI: 1.32–5.27, *P* = 0.001). Weight loss is a core nutritional feature in pancreatic cancer, mainly driven by malabsorption secondary to PEI and tumor-related metabolic disorders (33, 34). The observed difference in weight maintenance may be linked to meal-specific dose titration and administration with meals, which reduces premature enzyme inactivation and synchronizes digestion with nutrient intake, thereby accompanying better fat and protein absorption and relieving negative energy balance. This finding aligns with previous studies demonstrating that optimized PERT adherence correlates with weight outcomes in pancreatic cancer patients (35). However, our study extends these observations by quantifying the preservation of specific body composition compartments—skeletal muscle and adipose tissue—via CT-based analysis. Beyond statistical significance, the observed difference of 3.29 kg (5.37%) in weight preservation between the two groups holds clinical relevance in the context of pancreatic cancer. Several lines of evidence support this assertion. First, according to the international consensus definition, unintentional weight loss exceeding 5% within the preceding 6 months is a validated diagnostic criterion for cancer cachexia (36, 37). In our study, the Standard PERT group reached this threshold (6.90% loss) within a much shorter timeframe of only 30 days, whereas the Individualized PERT group (1.53% loss) remained below the diagnostic cutoff. Second, preoperative weight loss exceeding 5%–10% has been consistently associated with increased postoperative complication rates, prolonged hospital stay, and reduced tolerance to adjuvant chemotherapy in pancreatic cancer patients (38, 39). Therefore, the 3.29 kg difference observed in our study—equivalent to approximately 5%–6% of body weight in an average patient—may reflect clinically meaningful benefits in terms of chemotherapy completion rates, treatment-related toxicity, and potentially overall survival. While these conjectures require prospective validation, the observed weight differences associated with individualized PERT (3.29 kg, 5.37%) highlight its potential clinical relevance beyond mere statistical significance.

Secondary endpoints including serum ALB, body composition indices, immune - inflammatory biomarkers, and clinical outcomes further verified the favorable correlative profiles of individualized PERT. For serum ALB, the Individualized PERT group showed a smaller reduction than the Standard PERT group, although the between-group difference did not reach statistical significance (*P* = 0.110); however, the direction of effect favored the individualized PERT. This may be partly explained by albumin’s role as a negative acute-phase protein, whose synthesis is suppressed during postoperative inflammation (40). However, this null finding should be interpreted cautiously and warrants confirmation in larger prospective studies. After multivariable adjustment, the individualized PERT still showed a favorable trend for albumin, suggesting a potential nutritional pattern masked by the acute inflammatory response.

Body composition analysis confirmed that the individualized PERT was associated with milder loss of skeletal muscle mass (SMA, SMI) and adipose tissue (SAT, VAT) compared with the Standard PERT group. Preservation of skeletal muscle is an established prognostic factor in pancreatic cancer and is associated with better postoperative recovery and chemotherapy tolerance (41, 42). These results suggest that the observed weight difference reflected genuine retention of functional tissues rather than fluid accumulation.

Immune-inflammatory analysis further showed that LBC and LCR were more stable in the Individualized PERT group, while this group presented significantly lower levels of CRP, CAR, and LCS. These changes indicate milder systemic inflammation and relatively preserved adaptive immune function. This inter-group difference may also be linked to better intestinal barrier function and reduced bacterial translocation secondary to enhanced nutrient absorption (43, 44). Emerging evidence suggests that PEI is associated with gut microbiome dysbiosis and bacterial translocation, which may contribute to systemic inflammation. By optimizing fat digestion, individualized PERT has been shown to correlate with altered gut microbiota composition and promote the colonization of beneficial bacteria in animal studies (44, 45); whether this translates into reduced postoperative inflammation in humans warrants further investigation.

In terms of safety, no significant differences were observed between groups in the overall rate of postoperative complications or 30-day readmission rates. These findings support the clinical safety and feasibility of dietitian-led individualized PERT in perioperative care for pancreatic cancer patients.

The strengths of this study include a relatively large real-world sample, objective body composition assessment using L3 - level CT imaging, a comprehensive panel of immune-inflammatory biomarkers, rigorous multivariate adjustment for major confounders, and a clinically replicable dietitian-led individualized PERT protocol. These features enhance the reliability and translational value of the results.

Nevertheless, this study has several important limitations. First, this is a single-center, retrospective, non-randomized cohort study, which is inherently susceptible to selection bias, indication bias, and residual confounding. Therefore, causal relationships cannot be established, and the present results should be regarded as observational and hypothesis-generating rather than definitive evidence of efficacy. Second, given the retrospective design of this study, standardized quantitative data for grading PEI severity were not fully available from archived medical records. Accordingly, PEI diagnosis was determined using a combined approach incorporating clinical symptoms, documented unintentional weight loss, and clinical response to PERT. This diagnostic strategy is consistent with routine real-world clinical practice; however, it prevents us from performing subgroup analyses stratified by PEI severity. Third, the single-center design and relatively small sample size limit the generalizability of the conclusions. Fourth, follow-up was limited to the short-term period before initiation of adjuvant chemotherapy; long-term outcomes including survival, chemotherapy tolerance, and quality of life were not evaluated. Finally, unmeasured confounders including PERT adherence and actual intake of energy, protein and fat may have influenced the findings. Although all patients received standardized recipe guidance and generally maintained good compliance, objective quantitative records could not be obtained in this retrospective analysis. We will fully monitor these indicators in future prospective studies.

Within the limitations of this single-center retrospective cohort study, these findings suggest that dietitian-led individualized PERT combined with nutritional counseling is independently associated with more favorable short-term nutritional status, body composition, and immune-inflammatory balance in postoperative pancreatic cancer patients with PEI without compromising safety. These hypothesis-generating observations support further prospective, randomized, controlled trials to confirm the efficacy, optimal implementation, and long-term impact of this diet-led intervention, including long - term outcomes such as survival, chemotherapy tolerance, and quality of life.

## Data Availability

The raw data supporting the conclusions of this article will be made available by the authors, without undue reservation.
