# Supplementary material for: Dietitian-led individualized pancreatic enzyme replacement therapy is associated with favorable nutritional and immune-inflammatory profiles in pancreatic cancer: a retrospective cohort study
Source: Front Nutr. 2026 Jul 21;13:1683101. doi: 10.3389/fnut.2026.1683101 (PMC13435668; doi:10.3389/fnut.2026.1683101)
Supplement: Supplementary file 1 [file Table_1.docx]

**Supplementary Appendix**

**Supplementary Table 1. Adjusted associations between individualized PERT and outcomes**

| **Outcome Variable** | **Statistical Model** | **Adjusted Effect Estimate (95% CI)** | **Std. Error** | **t/Wald χ^2^** | ***P*** |
| --- | --- | --- | --- | --- | --- |
| Weight change (kg) | Multiple Linear Regression | β = 3.65 (1.57 ~ 5.73) | 1.05 | 3.47 | **＜0.001** |
| Weight change percentage(%) | Multiple Linear Regression | β = 5.72(2.48 ~ 8.96) | 1.64 | 3.50 | **＜0.001** |
| BMI change (kg/m²) | Multiple Linear Regression | β = 1.36 (0.56 ~ 2.15) | 0.40 | 3.38 | **＜0.001** |
| SMI change (cm²/m²)^*^ | Multiple Linear Regression | β = 6.85(5.24 ~ 8.45) | 0.81 | 8.45 | **＜0.001** |
| CAR change | Gamma GLM (Log Link) | Exp(B) = 0.50 (0.34 ~ 0.74) | 0.20 | 12.22 | **＜0.001** |
| LCR change | Gamma GLM (Log Link) | Exp(B) = 2.52 (1.10 ~ 5.75) | 0.42 | 4.81 | **0.028** |

All models were adjusted for tumor stage (≤1 vs. >1), baseline HB and SMI; models marked with * were adjusted for tumor stage and baseline HB only. For Gamma GLM, Exp(β) > 1 indicates higher levels in the Standard PERT group (reference). *P* < 0.05 results are in bold. Abbreviations: CI, confidence interval; BMI, body mass index; SMI, skeletal muscle index; CAR, C-reactive protein-to-albumin ratio; LCR, lymphocyte - C - reactive protein ratio.
