# Supplementary material for: Dietitian-led individualized pancreatic enzyme replacement therapy is associated with favorable nutritional and immune-inflammatory profiles in pancreatic cancer: a retrospective cohort study
Source: Front Nutr. 2026 Jul 21;13:1683101. doi: 10.3389/fnut.2026.1683101 (PMC13435668; doi:10.3389/fnut.2026.1683101)
Supplement: Supplementary file 2 [file Table_2.docx]

**Supplementary Table 2. Changes in body composition indices from baseline to endpoint**

| **Variables** | **Time point** | **Standard PERT group (n = 65)** | **Individualized PERT group**  **(n = 58)** | **Mean / Median difference** | **Difference (95% CI)** | **t / Z - value** | ***P*-value** |
| --- | --- | --- | --- | --- | --- | --- | --- |
| **SMD (HU)** |  |  |  |  |  |  |  |
|  | Baseline | 30.01±9.61 | 32.25±11.49 | 2.24 | (-1.52 ~ 6.00) | 1.18 | 0.240 |
|  | Endpoint | 30.19±8.50 | 30.35±10.23 | 0.15 | (-3.18 ~ 3.49) | 0.09 | 0.928 |
|  | Δvalue | 0.19±5.83 | -1.90±5.55 | -2.09 | (-4.11 ~ -0.06) | -2.04 | 0.043 |
| **SMA (cm²)** |  |  |  |  |  |  |  |
|  | Baseline | 104.43±24.10 | 111.19±25.14 | 6.76 | (-1.99 ~ 15.52) | 1.53 | 0.129 |
|  | Endpoint | 86.95±16.54 | 112.90±20.25 | 25.95 | (19.40 ~ 32.51) | 7.84 | ＜0.001 |
|  | Δ value | -17.48±13.97 | 1.71±11.47 | 19.19 | (14.63 ~ 23.75) | 8.33 | ＜0.001 |
| **SMI (cm²/m²)** |  |  |  |  |  |  |  |
|  | Baseline | 37.58±6.71 | 40.28±7.43 | 2.71 | (0.19 ~ 5.22) | 2.13 | 0.035 |
|  | Endpoint | 31.39±4.61 | 40.93±5.28 | 9.55 | (7.79 ~ 11.30) | 10.75 | ＜0.001 |
|  | Δ value | -6.19±4.72 | 0.65±4.07 | 6.84 | (5.27 ~ 8.41) | 8.62 | ＜0.001 |
| **SAT (cm²)** |  |  |  |  |  |  |  |
|  | Baseline | 99.78(68.48,123.72) | 97.32(59.39,143.53) | 1.58 | (-17.40 ~ 21.46) | -0.15 | 0.879 |
|  | Endpoint | 70.28(47.66,94.82) | 82.12(62.85,123.98) | 16.15 | (1.08 ~ 30.50) | -2.06 | 0.039 |
|  | Δ value | -16.40(-36.96,-2.26) | -2.28(-17.52,7.33) | 13.64 | (4.88 ~ 21.68) | -3.16 | 0.002 |
| **VAT (cm²)** |  |  |  |  |  |  |  |
|  | Baseline | 60.62(33.26,94.56) | 65.50(28.43,109.81) | 3.31 | (-14.34 ~ 20.74) | -0.43 | 0.671 |
|  | Endpoint | 46.58(18.92,71.10) | 62.58(29.40,90.04) | 14.17 | (2.36 ~ 27.46) | -2.34 | 0.020 |
|  | Δ value | -15.32(-34.58,3.40) | -1.94(-20.28,6.84) | 11.62 | (2.16 ~ 21.06) | -2.43 | 0.015 |
| **L3 vertebral body density (HU)** |  |  |  |  |  |  |  |
|  | Baseline | 194.57±70.19 | 190.14±58.10 | -4.42 | (-27.41 ~ 18.57) | -0.38 | 0.704 |
|  | Endpoint | 190.04±63.55 | 191.17±55.03 | 1.13 | (-20.07 ~ 22.32) | 0.11 | 0.916 |
|  | Δ value | -4.53±50.80 | -2.29±41.50 | 2.24 | (-14.31 ~ 18.79) | 0.27 | 0.789 |

Normal distribution data presented as mean ± standard deviation (SD); between-group difference reported as mean difference with 95% CI of mean difference; non-normal distribution data presented as median (interquartile range, IQR); between-group difference reported as median difference with 95% CI for median; *P*-value for between-group comparison of changes. Abbreviations: CI, confidence interval; SMD, skeletal muscle density; SMA, skeletal muscle area; SMI, skeletal muscle index; SAT, subcutaneous adipose tissue area; VAT, visceral adipose tissue area.Δ value, the difference between

the endpoint value and baseline value for each variable.
