# Supplementary material for: Dietitian-led individualized pancreatic enzyme replacement therapy is associated with favorable nutritional and immune-inflammatory profiles in pancreatic cancer: a retrospective cohort study
Source: Front Nutr. 2026 Jul 21;13:1683101. doi: 10.3389/fnut.2026.1683101 (PMC13435668; doi:10.3389/fnut.2026.1683101)
Supplement: Supplementary file 3 [file Table_3.docx]

**Supplementary Table 3. Changes in inflammatory biomarkers from baseline to endpoint**

| **Variables** | **Time point** | **Standard PERT group (n = 65)** | **Individualized PERT group**  **(n = 58)** | **Mean / Median difference** | **Difference (95% CI)** | **t / Z - value** | ***P*-value** |
| --- | --- | --- | --- | --- | --- | --- | --- |
| **CRP (mg/L)** |  |  |  |  |  |  |  |
|  | Baseline | 3.50(2.30,9.30) | 3.55(2.53,6.78) | -0.20 | (-1.10 ~ 0.80) | -0.34 | 0.738 |
|  | Endpoint | 30.40(7.50,66.10) | 20.37(2.85,38.99) | -9.30 | (-20.98 ~ -0.98) | -2.38 | 0.018 |
|  | Δ value | 18.60(1.20,53.60) | 13.54(-1.08,29.76) | -8.76 | (-20.38 ~ 0.36) | -1.85 | 0.065 |
| **PCT (ng/ml)** |  |  |  |  |  |  |  |
|  | Baseline | 0.10(0.10,0.10) | 0.10(0.10,0.10) | 0.00 | (0.00 ~ 0.00) | -1.43 | 0.153 |
|  | Endpoint | 0.10(0.06,0.15) | 0.10(0.06,0.10) | 0.00 | (-0.02 ~ 0.00) | -0.83 | 0.404 |
|  | Δ value | 0.00(-0.01,0.04) | 0.00(-0.03,0.02) | 0.00 | (-0.02 ~ 0.01) | -0.25 | 0.799 |
| **IL-6 (pg/ml)** |  |  |  |  |  |  |  |
|  | Baseline | 1.00(1.00,1.00) | 1.00(1.00,1.00) | 0.00 | (0.00 ~ 0.00) | -0.29 | 0.770 |
|  | Endpoint | 12.98(1.00,26.13) | 9.90(1.00,27.98) | 0.00 | (-5.05 ~ 2.86) | -0.36 | 0.717 |
|  | Δ value | 9.06(0.00,25.13) | 7.05(0.00,25.32) | 0.00 | (-4.69 ~ 2.32) | -0.21 | 0.834 |
| **CAR** |  |  |  |  |  |  |  |
|  | Baseline | 0.09(0.06,0.24) | 0.09(0.06,0.17) | -0.05 | (-0.29 ~ 0.02) | -0.41 | 0.685 |
|  | Endpoint | 0.86(0.24,2.03) | 0.55(0.07,1.07) | -0.34 | (-0.71 ~ -0.05) | -2.75 | 0.006 |
|  | Δ value | 0.61(0.03,1.50) | 0.39(-0.03,0.88) | -0.29 | (-0.65 ~ -0.02) | -2.11 | 0.035 |
| **LMR** |  |  |  |  |  |  |  |
|  | Baseline | 4.00(2.75,5.00) | 4.00(2.69,5.63) | 0.11 | (-0.50 ~ 0.73) | -0.39 | 0.698 |
|  | Endpoint | 1.89(1.17,4.00) | 1.64(1.11,2.75) | -0.21 | (-0.67 ~ 0.20) | -1.08 | 0.281 |
|  | Δ value | -1.75(-2.50,0.00) | -1.91(-3.57,-0.62) | -0.45 | (-1.26 ~ 0.26) | -1.25 | 0.212 |
| **LCS** |  |  |  |  |  |  |  |
|  | Baseline | 1.00(0.00,1.00) | 1.00(0.00,1.00) | 0.00 | (0.00 ~ 0.00) | -0.67 | 0.505 |
|  | Endpoint | 1.00(1.00,2.00) | 1.00(1.00,1.00) | 0.00 | (-1.00 ~ 0.00) | -2.18 | 0.029 |
|  | Δ value | 1.00(0.00,1.00) | 0.00(-0.75,1.00) | 0.00 | (-1.00 ~ 0.00) | -2.03 | 0.042 |

Normal distribution data presented as mean ± standard deviation (SD); between-group difference reported as mean difference with 95% CI of mean difference; non-normal distribution data presented as median (interquartile range, IQR); between-group difference reported as median difference with 95% CI for median; *P*-value for between-group comparison of changes. Abbreviations: CI, confidence interval; CRP, C-reactive protein; PCT, procalcitonin; IL-6:Interleukin-6; CAR, C-reactive protein - to -albumin ratio; LMR, lymphocyte-to-monocyte ratio; LCS, lymphocyte CRP score;Δvalue, the difference between

the endpoint value and baseline value for each variable.
